# When patients decide the admission – a four year pre-post study of changes in admissions and inpatient days following patient controlled admission contracts

**DOI:** 10.1186/s12913-020-05101-z

**Published:** 2020-03-18

**Authors:** Olav Nyttingnes, Torleif Ruud

**Affiliations:** 1 R&D Department, Division of Mental Health Services, Akershus University Hospital, PB 1000, 1478 Lørenskog, Norway; 2 Health Services Research Unit, Akershus University Hospital, Akershus, Norway; 3 Institute of Clinical Medicine, University of Oslo, Oslo, Norway

**Keywords:** Mental health care, Inpatient care, Pre-post study, Patient autonomy, User participation, Severe mental disorders, Admission procedures

## Abstract

**Background:**

Mental health professionals usually decide patients’ access to inpatient care to ensure the rational and fair distribution of care based on need and prognosis. The purpose of the current study is to investigate the effects of increasing patients’ influence on admission by enabling patients to initiate brief inpatient stays of up to five days at a community mental health center. Patients can initiate admission according to their own discretion, outside the existing referral and gatekeeping system.

**Methods:**

Patient-controlled admission (PCA) contracts were offered to eligible patients for inpatient stays in four community mental health centers in one health trust in Norway. Data on included patients’ inpatient stays at any of the hospitals’ mental health or addiction wards were collected by hospital electronic journal data extraction specialists for the two years before PCA contracts were introduced and the first two years after PCA contracts were introduced for the included patients.

**Results:**

The included patients (*n* = 57) had 406 PCAs in the two years following signing PCA contracts. When comparing the periods before and after the introduction of the contracts, the total number of admissions increased from 203 to 498 (*p* < .001), while the number of inpatient days decreased from 7172 to 3178 (*p* < .001). No significant change in involuntary care was observed. A comparison of box plots of inpatient day use in the eight half-year periods of the study indicates a gradual increase in median inpatient days up to the signing of a PCA contract for the sample, and an abrupt reduction to a stable median level of inpatient days after signing a contract.

**Conclusions:**

The included patients’ use of inpatient days changed profoundly after signing PCA contracts, similar to what previous studies of PCAs have indicated. In spite of the marked reductions in inpatient days, the pre-post design makes it impossible to rule out that the reductions were caused by regression toward the mean. No study of PCAs has reported negative effects, indicating that giving patients control over very short admissions is a feasible and potentially positive scheme in mental health care wards.

## Background

Throughout the history of psychiatry, access to inpatient care has usually been decided by professional mental health experts, striving to secure the always limited capacity for those with the greatest need and best prognosis. While professional control of access to care seems inevitable, there are also negative aspects to this gatekeeping, such as the risk of failing to admit patients early in a deteriorating process and learned helplessness in the patient [1]. Also, persons may act strategically in relation to gatekeepers to achieve their personal aims, be it access to care or avoidance of care. Adding coercion and involuntary care to the provider’s toolbox increases the expected complexity of strategic behaviors in persons with severe mental disorders, as well as the complexity of properly interpreting symptoms and severity.

Patients and staff sometimes relate case histories to illustrate the benefits of arranging a scheme for readmission based on the patient’s own evaluation of need. One example is a published case report where a young man diagnosed with schizophrenia and substance abuse was given an extensive outpatient care arrangement, supplemented by a symbolic voucher for five inpatient days over 12 months. The arrangement seemed to work well, giving the patient access to some inpatient days, while stimulating continued improvement in the community [2]. A review of brief admissions for unstable personality disorders has also reported promising results for involving patients in the goals of brief admission schemes [3].

In 2005, the Jæren Community Mental Health Centre (CMHC) in South-Western Norway invented, introduced, and studied the effect of patient-controlled admission (PCA) contracts. This took place in an inpatient ward working with the rehabilitation of schizophrenia and related conditions. The ward had seen overcrowding and many involuntary admissions, staff felt worn out and unsafe, and wanted to improve the usefulness of admissions. The ward decided to redistribute some power to the patients, but under substantial internal doubt and disagreement [4]. Patients well known in this ward who signed the contract were given access to short self-initiated admissions at the CMHC. The aim was to help them through an episode of deterioration without having to consult the GP or go to the emergency room. A PCA could be initiated simply by calling the ward and asking for it, thereby bypassing the traditional gatekeeping of admission by health and mental health experts. The PCA stays had to be as short as possible, at a maximum of five days, followed by a quarantine period of 14 days before another PCA was possible. Patients had to stay in the ward until discharge, and treatment ingredients, such as medication or the outpatient care plan were not supposed to be changed during a PCA. Being under a community treatment order (CTO) did not exclude the possibility of a PCA, and a voluntary self-referred admission would be counted as a voluntary admission in the national statistics, regardless of CTO status. The ward reserved two beds for PCAs. As expected, patients initiated many PCAs, so the total number of admissions for these patients increased greatly (by 2.6 times). Nevertheless, in the mirror-image evaluation study, comparing an average contract period of 19 months with similar pre-contract periods for each patient, the number of inpatient weeks was reduced by 33% after signing a PCA contract. The number of weeks under involuntary admission was reduced even more, by 43% [5]. With PCAs, it seemed possible to combine increased user influence with reduced inpatient days and days under coercion for patients with schizophrenia spectrum disorders. As a result, the paper caught the interest of the Norwegian health authorities, user organizations and hospitals, and similar arrangements were evaluated in two small similar studies in the following years [6, 7].

These initial findings were reported in Norwegian and partly in the grey literature, thus precluding attention outside the Scandinavian language area. A systematic review by Strand and von Hausswolff-Juhlin [1] presented the scheme in English, and concluded that, up to then, all PCA literature was in Norwegian, offered low-quality evidence in the form of pre-post design, and indicated similar promising reductions in inpatient days and involuntary care, as described above.

Later, PCAs were studied in a small randomized controlled trial from middle Norway. In the year after the introduction of PCA contracts, patients used PCAs to a limited degree (with a median of 1.5 times and five days); nevertheless, both the control and the intervention group had reduced their use of inpatient days by almost 45% from a high level of days (113 and 140 days, respectively), and no important effect differentiated the intervention group from the control group [8]. In a larger Danish study, 422 patients used 1037 PCAs (with a mean of 2.5) in the year following the signing of PCA contracts, and the reduction in inpatient days from the previous year was 17. The study found no important differences between PCA patients and a propensity-matched, register-based sample of 2110 patients drawn from all Danish mental health inpatients in the same period [9].

These results suggest that the promising results reviewed earlier [1] may be due to regression toward the mean. The PCA scheme is well suited to include patients with a high use of inpatient days and good cooperation with the ward, with a potential for improvement and an increased internal locus of control. Such patients can be likely to reduce inpatient stay regardless of PCA or not. These later studies nevertheless report a somewhat restricted use of PCAs, which may indicate issues in the implementation of the PCA scheme.

Another important aspect of the scheme is the preferences of patients and family carers. In an interview evaluation of the Jæren PCA scheme, patients expressed strong satisfaction with the freedom, safety and control provided by PCAs. Patients with PCAs appreciated sparing referral and admission interviews with general practitioners (GP), the emergency unit or the emergency/acute ward, and getting help before serious deterioration. Even patients who had not initiated PCAs felt safer with the PCA option. Municipal care personnel strongly endorsed the PCA scheme, and noted that PCAs improved the patients’ relations with the ward personnel and the family carers’ confidence in mental health services. The ward staff, who had been partly skeptical about PCAs, were surprised by how well patients coped with the scheme. The focus on rest and shielding from crisis by restricting tours and leaves of absence sometimes caused patients to discharge themselves as early as after one or two days, and patients and family carers also voiced some criticism of the quarantine and too few days allowed under a PCA [4]. Patients on PCAs tended to report increased patient autonomy and agency [10]. Similar findings were later reported in Denmark: Patients signed PCA contracts to get access to early help, avoid admissions at emergency units, and avoid getting very ill or having a long admission. The PCAs were initiated after an increase in symptoms, as well as following social or practical problems, or to spare family carers [11]. These findings imply that PCAs can be a relevant measure as long as it shows non-inferiority for other variables.

Akershus University Hospital was inspired by Jæren’s PCA scheme, and ran a pilot project introducing PCA in one CMHC with positive results [6]. When introducing the PCA scheme to all four CMHCs, we decided to evaluate the scheme in a pre-post study with a larger sample and a longer observation period, to be able to better evaluate whether all effects of PCA were due to regression toward the mean.

The aims of this study were to examine the following research questions:

- What are the main patterns of PCA utilization?
- Is the patients’ use of inpatient days reduced in the two years after signing a PCA contract compared to the two years before the introduction of a contract?
- Are the patients’ days under involuntary inpatient care reduced after signing the PCA contract?
- Are there subgroups, such as main diagnostic groups, with relatively larger inpatient day reductions?
- Can the pattern of changes during the four-year observation period be explained solely by regression toward the mean?

## Methods

### The study context

In Norway almost all mental health services are public, with small user fees for outpatient consultations and no fees for inpatient services. At the time of the present study, there were 79 mental health beds per 100,000 adult inhabitants [12]. Adult mental health specialist services are divided into hospital services and CMHCs. Hospital services typically consist of acute wards, a combined high security and forensic unit, addiction wards and psychogeriatric inpatient services. The CMCHs typically have general mental health outpatient clinics, open-door inpatient wards, specialist teams and ambulant services. In 2014, 41% of mental health inpatient capacity (beds) was located in CMHCs [13]. Akershus University Hospital serves a catchment area of circa 500,000 inhabitants, with hospital services as described above and four CMHCs. The PCA scheme had been utilized at two CMHCs before the project started.

### Intervention

In the Akershus University Hospital PCA study, PCA contracts were offered to patients with content and inclusion criteria as described in the introduction. Each ward reserved two beds for PCAs. The patient should be well known in the ward and with a recent history of admissions to inpatient mental health care, and PCA had to be considered by a psychiatrist or psychologist to be a good solution for the patient. With some exceptions, patients with severe addiction problems were not offered PCA. A call for PCA could be made on any weekday between 09:00 and 20:00. If the dedicated PCA beds were occupied, the patient was asked to call back later or to initiate a standard referral procedure.

### Design

The current study is a pre-post study, also called a mirror-image study, where we compare the outcome variables for equal time periods before and after the signing of PCA contracts. The included patients thus serve as their own control. Because regression toward the mean is a common source of error in pre-post studies, we decided to study inpatient days and involuntary inpatient days during the two years before and two years after the event, to see whether the periods immediately before inclusion had a higher utilization of inpatient services than the previous periods. Patients signing a contract after recruitment started were asked for consent to participate in the Akershus University Hospital PCA study. Patients with a PCA contract at any time during the last two years could not be included in the current study. The primary outcome measure was inpatient days, as recorded in the electronic patient journal of Akershus University Hospital before and after signing the PCA contract.

### Measurements

Staff at the CMHC recorded patient characteristics at inclusion for patients giving informed written consent to participate in the study. These data included gender, age, living situation, main ICD-10 diagnosis [14], Health of the Nation Outcome Scales (HoNOS) for adults [15], and use of alcohol and drugs in the previous six months [16]. Following each request for a PCA, staff filled in a form with information on the request, whether the patient had been offered PCA or not, and if not, the reason for this.

The primary outcome measure was inpatient days, and secondary outcome measures were admissions and involuntary inpatient days. The patients’ use of any mental health inpatient care was collected by the hospital’s electronic patient journal data extraction specialists. For each patient, the four-year study period was split into eight half-year periods. Admissions were assigned to the six-month period relative to the inclusion when the first day of the admission took place. Inpatient days were assigned to the period when they took place, such that days in one admission could have the days split between different six-month periods. For admissions that included transfer between wards, the admission was assigned to the first ward of the stay, while days were allocated to the wards where the patient stayed.

### Sample

Four CMHCs recruited patients, and the numbers of patients declining to sign the PCA contract or participate in the research were not recorded. During 2011 and 2012, 64 patients were recruited for the study. At the end of the study period, electronic information on admissions was unavailable for six patients, who were then excluded from the study. One patient had such a large number of inpatient days during the two years before signing the PCA contract, that it was untypical for the sample and for current Norwegian mental health care. We considered this as outlying data which would influence the results too much, and therefore excluded this patient from the analyses below.

### Statistical analyses

Previous PCA studies did not report standard deviations, but we expected great variation in reductions between patients. Even when allowing a standard deviation twice the size of the mean reduction, a sample size of 34 patients would obtain statistical power at the recommended level of .80 with alpha value of .05 [17]. Based on the inclusion time and capacity for PCA contracts, this sample size was attainable, and we sought to recruit eligible and consenting patients throughout the 24-month inclusion period.

We inspected the distributions of inpatient days per patient in the sample and compared inpatient days in each CMHC or hospital ward before and after the contract, and did the same for the number of inpatient days under involuntary care. The periods were compared using the Wilcoxon signed-rank test, because the samples were positively skewed. In order to illustrate and examine the detailed development of changes in inpatient days for the sample, we constructed box plots for each six-month period relative to the contract initiation.

We examined the effect of PCA on the mean and median reduction of inpatient days in subgroups of different diagnosis, HoNOS scores, alcohol and substance abuse, living alone or not, and use of municipal services. Due to the small subsamples, we did not conduct tests for equality of means, and the results should be considered as ideas for future studies.

## Results

The eligibility criteria and recruitment procedures resulted in the sample characteristics presented in Table 1. The sample had a majority of women, and half of the patients had a main diagnosis of psychotic disorders (F20–32) or an addiction disorder (F10–19). The HoNOS scores indicate that the most frequent problems in the sample were depressed mood, problems with relations to others, and other mental health problems, with 18 (75%) of these problems specified as anxiety.

**Table 1:** Patient characteristics at the time of inclusion (*N* = 57)

	Number	Percent
*Gender*		
Women	30	52.6
Men	26	45.6
Missing	1	1.8
*Age*		
20–29	7	12.3
30–39	14	24.6
40–49	16	28.1
50–59	15	26.3
60–66	4	7.0
Above 66	1	1.8
*Marriage/cohabitating*		
Married or living with a partner	10	17.5
Unmarried, widowed or divorced	47	82.5
*Living alone*		
Yes	41	71.9
No	15	26.3
Missing	1	1.8
*Housing*		
Ordinary housing	47	82.5
Housing with part-time supervision	4	7.0
Housing with full-time supervision	4	7.0
Missing	2	3.5
*Main diagnoses (ICD-10 codes*^*a*^*)*		
Addiction disorder (F10–19)	3	5.3
Schizophrenia spectrum disorder (F20–29)	16	28.1
Bipolar disorder (F30–31)	9	15.8
Depressive disorder (F32–33)	8	14.0
Anxiety and adjustment disorders (F41–43)	8	14.0
Personality disorder (F60–69)	10	17.5
Missing	3	5.3
*Alcohol use*		
Abstinent or non-detrimental use	39	68.4
Abuse or dependency	7	12.3
Missing	11	19.3
*Drug use*		
Abstinent or non-detrimental use	41	71.9
Abuse or dependency	3	5.3
Missing	13	22.8
*HoNOS*^b^*score 3 or 4 (need for intervention)*		
H01. Overactive, aggressive, disruptive or agitated behavior	2	3.5
H02. Non-accidental self-injury	9	15.8
H03. Problem drinking or drug-taking	7	12.3
H04. Cognitive problems	6	10.5
H05. Physical illness or disability problems	9	15.8
H06. Problems associated with hallucinations and delusions	11	19.3
H07. Problems with depressed mood	23	40.4
H08. Other mental and behavioral problems	24	42.1
H09. Problems with relationships	23	40.4
H10. Problems with activities of daily living	9	15.8
H11. Problems with living conditions	1	1.8
H12. Problems with occupation and activities	6	10.5
*Total*	57	100.0

^a^ICD-10: International Statistical Classification of Diseases and Related Health Problems, 10th Revision

^b^HoNOS: Health of the Nation Outcome Scale

### Patient-controlled admission utilization

PCAs were used extensively by the patients. For the 57 patients in the sample, we registered 247 (mean 4.3, median 3) requests for PCAs during the first year after the introduction of the PCA contract, and 229 (mean 4.0, median 3) in the second year. Of these 476 requests from 47 patients, 406 resulted in an admission. Fifty requests were declined due to a lack of available beds and five due to the quarantine. The mean duration of admissions that started as PCAs was five days. Twenty-eight PCAs (6.8%) ended up lasting longer than the contracted five days, and one of these was transferred from the PCA to an acute ward. For the 44 patients having more than one PCA, the mean and median time between the end of a PCA and the next request were 54 and 30 days, respectively (range 0–338 days, skewness 2.47).

### Change in admissions, inpatient days and involuntary care

The use of mental health inpatient days in the two-year periods before and after the PCA contract is presented in Table 2. All but one of the participants had used mental health inpatient services sometime during the two years before the introduction of the PCA contract. During these two years, the 57 participants used 7172 inpatient days (mean 125.8, s.d. 98.2, skewness 1.5) in 203 admissions (mean 3.6%, s.d. 2.9, skewness 3.0). During the two years *after* the introduction of the PCA contract, all but six of the patients used inpatient services in a total of 3994 inpatient days (mean 70.07, s.d. 62.83, skewness 0.58) and the number of admissions was 498. A Wilcoxon signed-rank test indicated that the reduction in inpatient days was significant (*Z =* − 3.74, *p* < .001. The average length of admission decreased from 35.33 days during the two years before the contract to 7.98 days in the two years after the contract. The reduction in the mean number of inpatient days from the year before the PCA contract to the year after the PCA contract was 46.7 inpatient days or 55.7% (from 83.8 to 37.1).

**Table 2:** Admissions and inpatient days, count and mean per patient

	Before PCA contract (24 months)			After PCA contract (24 months)			Difference in days	
Type of ward	Admissions^a^	Inpatient days	Mean days / patient (SD)	Admissions^a^	Inpatient days	Mean days / patient (SD)	Days	*P*-value^b^
Acute ward	63	815	14.3 (23.3)	36	218	3.8 (10.0)	597	< .001
High-security ward	1	527	9.2 (37.0)	1	198	3.5 (21.2)	329	.225
Addiction ward	2	33	0.6 (3.3)	1	7	0.1 (0.9)	26	.285
CMHC ward (PCA excluded)	137	5797	101.7 (81.1)	54	2142	37.6 (46.1)	2226^d^	< .001
PCA at CMHC ward	–	–		406	1429	25.1 (24.4)		
PCA request without admission	–	–		70	–			
Involuntary care	17^c^	435	7.6 (28.3)	14^c^	321	5.6 (26.6)	114	.397
Sum of admissions and days	203	7172	125.8 (98.2)	498	3994	70.1 (62.8)	3178	< .001

^a^ For admissions with internal transfers between wards, only the initial ward is counted, while the days are allocated to the ward type where the patient stayed

^b^*P*-values from Wilcoxon signed-rank tests

^c^ The number of admissions that included days under involuntary care

^d^ Difference between ordinary CMHC days before PCA contract and combined CMHC and PCA days after contract

In the two years before the introduction of the PCA contract, 13 patients had been involuntarily admitted for a total of 435 days. In the intervention period, four patients were under involuntary care for a total of 321 days. A Wilcoxon signed-rank test indicated that the reduction in involuntary days was not significant (*Z* = −.84, *p* = .40).

**Fig. 1:**
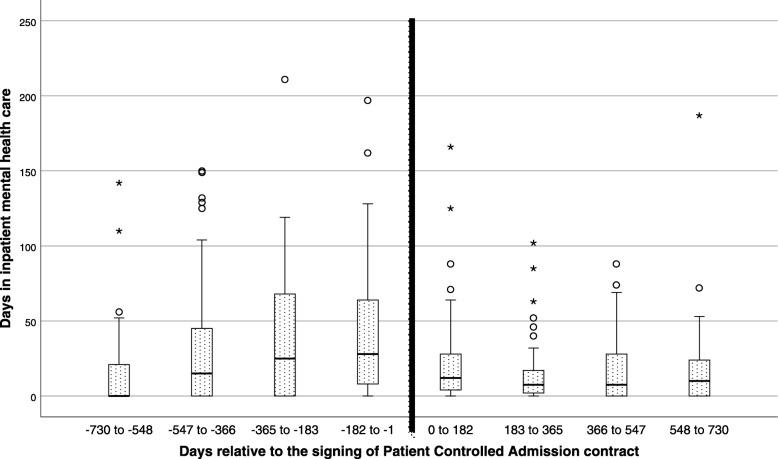
Box plot of inpatient days used, displayed as four six-month periods before and after the patients signed the PCA contract. Boxes represent the second and third quartiles, divided by the median bar. The whiskers mark an additional 1.5 interquartile range. Circles mark mild outlying observations (up to three interquartile ranges) and asterisks mark extreme outliers (outside three interquartile ranges) outside the middle quartiles

The pattern of changes in inpatient days over time relative to contract initiation is illustrated with the box plot for each six-month period in Fig. 1. For the first two of these half-year periods, as many as 35 and 26 patients did not use any inpatient days. During the last year before the contract, and in the two years after the contract, the number of patients not using inpatient care in any half-year period ranged from 11 to 22.

There was a profound change in the number and length of admissions following contract initiation: From the six months before to the first six-month period after PCA contracts, the number of admissions increased from 73 to 144, while the median (mean) number of days per admission changed from 19 (37.6) to 4 (8.8).

### Reductions in subgroups

To examine whether subgroups of patients differed in the effects of the PCA, we calculated the number of days spent in inpatient care, and examined the relative reduction in subgroups of patients according to diagnosis, alcohol and substance abuse, living alone or not, and use of municipal services.

Twenty-nine patients reduced their share of days spent as inpatients by more than 50%. The reductions in inpatient days for different subgroups of patients are presented in Table 3. Patients with a diagnosis of schizophrenia or with higher HoNOS score showed a high use of inpatient days before contract, and reductions in inpatient days of more than 90 days or 50%. Patients that did not use or receive municipal services tended to show greater reductions than those using those services. Patients diagnosed with bipolar disorder showed the smallest reduction in mean inpatient days; from 103 to 82. For 13 patients, the number of inpatient days increased between 14 and 150 days, but none of the recorded variables could predict increased inpatient day use. For this group, the mean number of inpatient days before contract (64.8; s.d. 37.9) was half of that of the other 44 patients (134.4, s.d. 92.3).

**Table 3:** Mean number of inpatient days in the two years before and after PCA contract initiation

Subgroup		Inpatient days		Reduction of days	
Diagnostic group	N	Before	After	Mean	Percent
Addiction disorder (F10–19)	3	107.0	30.3	76.7	71.7
Schizophrenia spectrum disorder (F20–29)	16	175.1	80.4	94.7	54.1
Bipolar disorder (F30–31)	9	102.7	82.2	20.4	20.0
Depressive disorder (F32–33)	8	105.6	68.8	36.8	34.8
Anxiety and adjustment disorders (F41–43)	8	128.1	80.8	47.4	36.9
Personality disorder (F60–69)	10	90.7	47.3	43.4	47.9
Missing	3	125.7	66.3	59.3	47.3
Severity of problems (HoNOS mean score)					
0–1 (lower tertial sum score)	18	111.9	65.0	46.9	41.9
1.1–1.3 (middle tertial sum score)	21	103.6	72.4	31.2	30.1
1.4–2.5 (upper tertial sum score)	18	167.2	72.0	95.2	56.9
Involuntary inpatient care in the two years before PCA contract					
1 or more involuntary inpatient days	13	177.8	100.2	77.5	43.6
No involuntary inpatient days	44	109.6	62.3	47.3	43.2
Alcohol use or drug abuse					
Abstinent	30	108.5	59.3	49.2	45.3
Use without problems	5	200.0	42.4	157.6	78.8
Abuse or dependency	9	145.7	66.1	79.6	54.6
Missing	13	125.7	107.8	17.9	14.2
Use of municipal day care center					
No	26	125.7	62.7	63.0	50.1
Yes	31	126.9	76.0	50.9	40.1
Conversations with municipal staff at least once per month					
No	17	147.5	57.1	90.4	61.3
Yes	40	117.3	75.4	41.9	35.7
Received municipal home services					
No	40	129.1	68.6	60.5	46.9
Yes	17	119.8	73.1	46.8	39.0
Outpatient consultation once per month or more					
No	35	122.2	63.8	58.3	47.8
Yes	22	132.9	69.9	53.3	47.4
Regular contact with psychosis treatment team					
No	42	114.9	69.6	45.2	39.4
Yes	15	158.4	70.7	87.7	55.4

## Discussion

The PCA scheme moves some gatekeeping powers from professional health experts to patients with mental disorders who have frequently required inpatient care. One could expect such a move from expert to lay decisions to result in low-quality admission decisions and overuse of mental health care. This study confirms that a PCA scheme, where patients are in control of very short admissions, is a feasible scheme in typically elective mental health wards where patients often have repeated admissions.

The sample was recruited by clinicians based on the stated aims of the PCA scheme. Comparing our sample to a national census of adult mental health inpatients from 2012 [18], both saw a slight female majority (53 vs 52%). Patients in our study were older with 12% below 30 years and 26% at 50–59 years, compared to 30% below 30 years and 14% at 50–59 years in the national sample. A comparable share of patients was living with a partner (18 vs 21%). Our sample had 44% with schizophrenia spectrum and bipolar disorders (ICD10 F20–31) compared to 39% at CMHCs in the national sample. The share of addiction disorders (F10-F19 was lower in our sample than the national sample from all kinds of inpatient units (5 vs 17%). This indicates that the selection of patients to PCAs with a sizeable need for help and an established relation to the CMHC resulted in fewer young patients, but with more severe mental disorders, and that patients with clear addiction problems were only occasionally offered PCA.

Comparing the two-year periods before and after the PCA contract, the number of admissions increased 2.5 times, combined with a reduction in average length of stay by more than 75%. The resulting reduction in inpatient days was 44.7%. These reductions are similar to several previous reports of PCA schemes [1], but much larger than those reported in Denmark [9]. Few patients in the current study had involuntary inpatient days and the dispersion in involuntary care before and after the PCA contract was large. The minor reduction found in involuntary days was not significant. Our findings of differing reductions in different subgroups must be considered tentative, as the subgroups are small, and several variables were available for analysis. The findings imply that PCA works somewhat better for patients with more severe problems, as indicated by a higher HoNOS score, a diagnosis of schizophrenia, or contact with a psychosis treatment team. Patients with less use of municipal services tended towards larger reductions. Perhaps the PCAs helped patients with severe problems and few municipal helpers around them to access inpatient care earlier in their deterioration process. Other possibilities are that longer admissions may effect community skills or support negatively, or that the shorter stays may improve upon learned helplessness or alliance, and that these effects may affect severe problems more profoundly.

When interpreting reduction in inpatient days in a pre-post study, an important concern is whether the observed changes are entirely due to regression toward the mean, or whether the intervention also played a role. The PCA scheme has high use of inpatient days as a criterion for a PCA contract, which increases the chance of recruiting patients when their usage of inpatient care peaks. When examining the box plots in Fig. 1, the reduction in inpatient day use following the PCA contract is substantial, and the new average level seems stable during the two years following the PCA contract. The median admission length changed from 19 to four days following the PCA contract, and this sharp drop indicates that the PCA process at least contributed to some form of change in patient behavior related to inpatient stays. However, there is also a clear pattern of a building up of inpatient days during the two years prior to the introduction of the PCA contract, where the first box plot indicates a median of zero, reflecting that 61% of included patients did not use any inpatient days in this period. Due to the nature of our data catchment of inpatient days, we do not know whether this reflects that the onset of disorder came later, that the patient had not yet moved to our catchment area, or simply a period of the disorder without need for inpatient care.

In our opinion, the issue of whether the reported effect of PCAs is entirely due to regression toward the mean remains unsettled. The results from the small randomized control trial in middle Norway reported a similar reduction in the control group as in the intervention group, indicating no true effect of PCA [8]. That study reported very high baseline levels of inpatient days in both control (a mean of 113 days) and intervention group (a mean of 141 days). All participating patients were informed of the scheme, and the control group patients were promised a contract after the trial period, so the control group might have been “contaminated” by the intervention. When the intervention group rarely used PCAs in that study, one can suspect that regression toward the mean from a very high level of inpatient days combined with changes introduced along with the PCA scheme for all patients are factors in the results. The larger Danish study reported some inequalities between the intervention and the register-based, propensity-matched control group [9]. More notably, the reduction of 17 inpatient days from pre to post was modest and each patient used 2.5 PCAs in the observation year, which constituted only half of the admissions for this group. In our current study, PCAs constituted more than 80% of the admissions in the two years after the intervention, and the mean number of inpatient days was reduced by 46 days from 83.8 days in the two one-year periods surrounding contract initiation. Also, the other admission types were substantially reduced in the intervention period: Acute/emergency and ordinary CMHC admissions were reduced by 42.9 and 60.6%, and inpatient days in these admissions were reduced by 73.3 and 63.0%, respectively. These details indicate that the PCAs may have worked or been implemented differently in these studies.

There are sound reasons for expecting different effects in different regions: When patients emphasize confidence in better access to inpatient care, the ability to admit following practical and social problems [4, 11] and boredom in wards [4], local variations in such factors during the baseline or intervention period may strongly influence results. The interview study from the initial PCA project at Jæren found that some patients did not use PCAs at all, while others only used them at the initiative of others. In addition, staff noted that some patients with a history of involuntary admissions had to learn that they could discharge themselves from PCAs without risk of retention [4]. In the Akershus University Hospital pilot study, some patients reported that PCAs reduced symptoms and made them feel safer, and thereby reduced the need for any form of inpatient care [6]. For patients who avoid mental health care in fear of retention, a PCA might change their coercion-avoiding strategies, and provide a care scheme where it is possible for them to use inpatient days more rationally and avoid deterioration of their conditions. Such patients might be outliers with a strongly positive effect of PCAs. The box plots indicate a strongly skewed distribution for inpatient days, and also note the excluded patient mentioned in the sample section. The frequency and intensity of all these aspects of care are unlikely to be equal between sites, and therefore the consequences and effects of PCAs are unlikely to be the same, even if the implementation of PCAs could be equal.

Patients who gained gatekeeping powers through PCAs have had a history of admissions that are usually set off by behavior interpreted as deterioration of rationality, insight and coping ability. It is noteworthy that no PCA study has reported any significant negative effect of signing PCAs. This indicates that the mental health patient’s private considerations and priorities are more important assets in gatekeeping than is commonly acknowledged. The observed effects range from the neutral to the strongly positive, supporting a non-inferiority evaluation of the PCA scheme.

### Strengths and limitations

The study collected data for a four-year period from four different CMHCs, and the results cannot be attributed to a single enthusiastic clinical milieu. Data on inpatient stays were collected from electronic patient records, and are likely to have high completeness. Having data from four years facilitated a more thorough consideration of the extent of regression toward the mean, and the durability of the results.

A main limitation is the pre-post design, which prevents conclusions regarding cause and effect. Patients were recruited based on clinical considerations of suitability for PCAs, which increases the risk of recruitment near a peak in inpatient days. We used only historical controls in our study. A recommendation for future pre-post studies is to add a propensity-matched sample as a control group to help disentangle regression toward the mean from the effect of the intervention. The modest sample size and lack of pre-specified hypothesis make findings on subgroups tentative. While the data collection procedure provided data for four years, lack of data catchment details prevented us from discovering the reasons why a majority of patients did not use any inpatient days in the first parts of the observation period. The study is from one health trust in an affluent country, and results cannot be generalized to other catchment areas without substantial qualifications.

## Conclusion

The current study revealed immediate changes and large reductions in length of stay and use of inpatient days for patients that signed a PCA contract. In spite of these major changes, the pre-post design cannot rule out that all inpatient reductions can be attributed to regression toward the mean. The combined results from studies of PCAs still indicate that planned and systematized patient influence over admissions is a promising approach. The lack of reported negative effects from PCAs questions the ubiquity of professional clinical judgement in the gatekeeping process, and suggests that patient autonomy within well-considered limits is an underutilized asset in mental health care. The observed effects may rest upon factors that may vary between sites and are difficult to generalize across sites, and PCAs and similar arrangements ought to be tested also outside of Scandinavia.

## Data Availability

The dataset analyzed in the current study are available from the corresponding author on reasonable request.

## Abbreviations

CMHC: Community mental health center
CTO: Community treatment order
GP: General practitioner
HoNOS: Health of the Nation Outcome Scales
ICD-10: International Statistical Classification of Diseases and Related Health Problems, 10th Revision
PCA: Patient-controlled admission
